# Forest malaria and prospects for anti-malarial chemoprophylaxis among forest goers: findings from a qualitative study in Lao PDR

**DOI:** 10.1186/s12936-021-04027-z

**Published:** 2022-01-05

**Authors:** Monnaphat Jongdeepaisal, Soulixay Inthasone, Panarasri Khonputsa, Vilayvone Malaphone, Kulchada Pongsoipetch, Tiengkham Pongvongsa, Mayfong Mayxay, Keobouphaphone Chindavongsa, Christopher Pell, Richard J. Maude

**Affiliations:** 1 Mahidol-Oxford Tropical Medicine Research Unit, Faculty of Tropical Medicine, Mahidol University, Bangkok, Thailand; 2 Centre for Tropical Medicine and Global Health, Nuffield Department of Medicine, University of Oxford, Oxford, UK; 3 Savannakhet Provincial Health Department, Savannakhet, Lao PDR; 4 Lao-Oxford-Mahosot Hospital-Wellcome Trust Research Unit (LOMWRU), Mahosot Hospital, Ministry of Health, Vientiane, Lao PDR; 5 Institute of Research and Education Development (IRED), University of Health Sciences, Ministry of Health, Vientiane, Lao PDR; 6 Center of Malariology, Parasitology, and Entomology, Vientiane, Lao PDR; 7 Amsterdam Institute for Global Health and Development (AIGHD), Amsterdam, The Netherlands; 8 Department of Global Health, Amsterdam University Medical Centers, Amsterdam, The Netherlands; 9 Centre for Social Science and Global Health, University of Amsterdam, Amsterdam, The Netherlands; 10 Harvard TH Chan School of Public Health, Harvard University, Boston, USA; 11 The Open University, Milton Keynes, UK

**Keywords:** Forest goer, Malaria, Forest, Intervention, Prophylaxis, Lao PDR

## Abstract

**Background:**

Despite significant decline in malarial incidence and mortality in countries across the Greater Mekong Subregion, the disease remains a public health challenge in the region; transmission continues mainly among people who visit forests in remote areas, often along international borders, where access to primary healthcare is limited. In the absence of effective vector-control measures and limited exposure periods, malaria chemoprophylaxis has been proposed as a strategy to protect forest goers. As a rarely used approach for indigenous populations, questions remain about its feasibility and acceptability. Drawing on in-depth interviews with forest goers and stakeholders, this article examines opportunities and challenges for implementation of anti-malarial chemoprophylaxis for forest goers in Lao PDR.

**Methods:**

In-depth interviews were conducted with 16 forest goers and 15 stakeholders in Savannakhet province, Lao PDR. Interview topics included experience of malaria prevention and health services, and perceptions of prophylaxis as a potential component of malaria elimination strategy. The interviews were transcribed and coded using inductive and deductive approaches for qualitative thematic analysis.

**Results:**

In ethnically and geographically diverse villages, awareness of malaria risk prompts forest goers to protect themselves, albeit sub-optimally using available preventive measures. Stakeholders highlighted challenges for targeting at-risk populations and approaches to address forest malaria in southern Lao PDR. Among policymakers, choice and cost of anti-malarials, particularly their efficacy and source of funding, were key considerations for the feasibility of malaria prophylaxis. Acceptability of prophylaxis among forest goers was also influenced by the complexity of the regimen, including the number of tablets and timing of doses. Implementation of prophylaxis may be affected by a lack of transportation and communication barriers in remote communities.

**Conclusion:**

Adding prophylaxis to existing malaria control activities requires strengthening the capacity of local health workers in Lao PDR. Ideally, this would be part of an integrated approach that includes strategies to address the other febrile illnesses that forest goers describe as priority health concerns. The prophylactic regimen also requires careful consideration in terms of effectiveness and simplicity of dosing.

**Supplementary Information:**

The online version contains supplementary material available at 10.1186/s12936-021-04027-z.

## Background

Malarial incidence and mortality have seen significant declines in countries across the Greater Mekong Subregion (GMS) over the past two decades [1]. Multiple key interventions, including better case detection and treatment, improvements in surveillance, stratification and targeting of residual transmission foci, are thought to have contributed to this progress [2, 3]. Nevertheless, the disease remains a public health challenge in the region with transmission continuing mainly in remote areas along international borders where access to primary healthcare is limited among people who visit forested areas, especially mobile and migrant populations. Moreover, faced with the threat of resistance to artemisinin-based combination therapy (ACT), countries in the GMS are making additional efforts to eliminate malaria by 2030.

In Lao PDR, the Center of Malariology, Parasitology, and Entomology (CMPE) has prioritized strategies to reduce malaria burden and interrupt transmission in focal areas across the country. Initiatives have included early diagnosis and treatment (EDAT) with rapid diagnostic tests (RDT) and ACT [4], providing vector control measures, and strengthening case management and disease surveillance [5]. Malaria burden, however, remains high in southern provinces where outbreaks occur, partly due to high mobility of cross-border populations and workers in industrial and agricultural plantations [5]. Although coverage of insecticide-treated nets (ITNs) has increased in these areas, their effectiveness is limited by the presence of outdoor biting vectors in forested settings, where people often spend time. Research into additional malaria control measures as part of a more tailored approach is needed to protect forest goers in this complex setting [6].

Anti-malarials have been used preventively in various transmission settings among at-risk groups where malaria burden is high. In Africa, the World Health Organization (WHO) recommends intermittent preventive treatment for pregnant women (IPTp) and for infants (IPTi) at scheduled antenatal or vaccination visits [7–9]. Seasonal malaria chemoprevention (SMC) has also been added as an additional prevention strategy for young children during the malaria transmission season in the Sahel [10]. To accelerate elimination, mass drug administration (MDA) has been successfully trialled, for example in the GMS, to clear the subclinical infections among populations in endemic areas [11, 12]. Chemoprophylaxis is recommended for international travellers and military personnel visiting endemic areas [13], but has not been recommended for indigenous populations, such as forest goers, in low transmission settings such as the GMS. In the absence of effective vector-control measures and limited periods of exposure, malaria prophylaxis has been proposed as a promising strategy to protect forest goers [14, 15].

Chemoprophylaxis for forest goers is currently under comprehensive clinical evaluation in Cambodia [16]. This approach could potentially overcome some of the barriers to effective malaria prevention for forest goers in Lao PDR and other areas of the GMS. However, additional information is needed to assess the feasibility of prophylaxis including whether forest goers would be willing to take anti-malarials when healthy, to what extent prophylaxis may influence other malaria prevention and control activities, and how it could be administered and managed by the health system.

Drawing on in-depth interviews with forest goers and stakeholders in endemic villages in Savannakhet province, this article examines the acceptability and feasibility of malaria prophylaxis for forest goers in Lao PDR. The article describes characteristics of forest goers and their experience with malaria infection, prevention, and treatment. Among forest goers and stakeholders, perceptions of implementing malaria prophylaxis for forest goers in the area are also hypothetically examined. The overall aim is to elaborate opportunities and challenges for implementation of malaria prophylaxis for forest goers in Lao PDR.

## Methods

### Setting

**Fig. 1:**
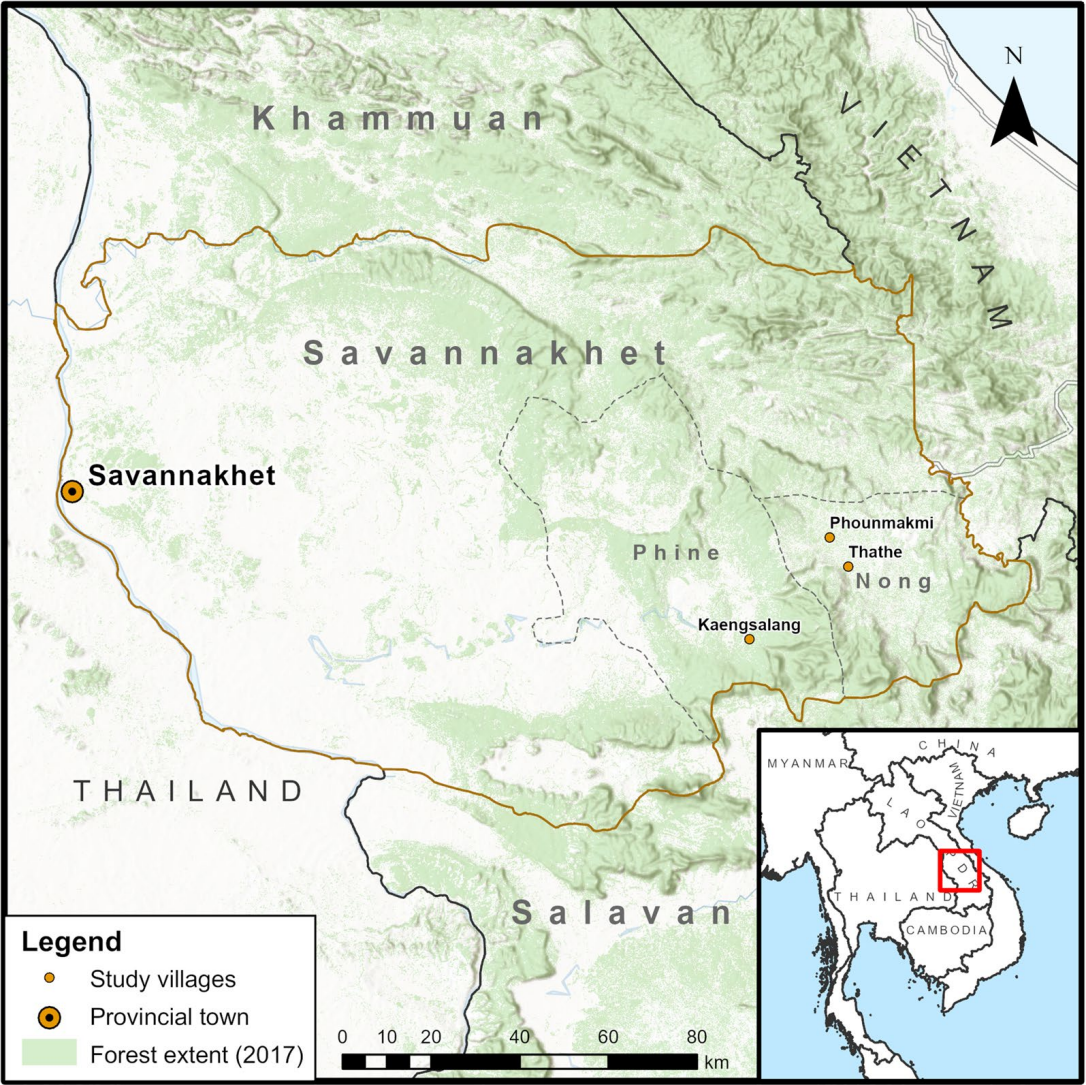
Map of study villages and districts

**Fig. 2:**
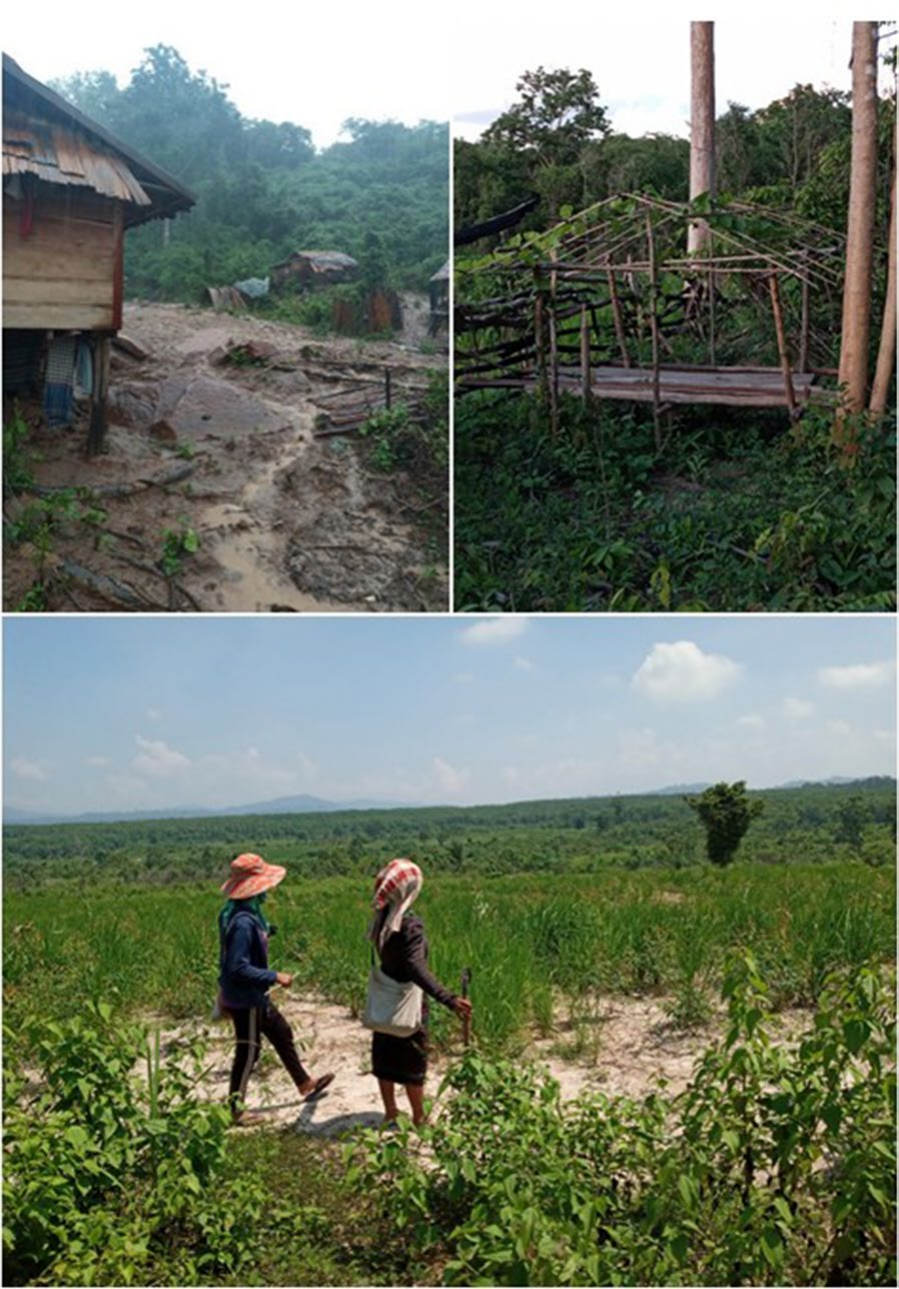
Livelihoods in study villages. **A** Village houses at foot of a mountain **B** Farm house or “Tieng na” close to the forest **C** Local residents in their farm clothing in rice field

The study was conducted in three malaria endemic villages in Nong and Phine districts, Savannakhet province, bordering Vietnam (Fig. 1). Farming and agricultural activities are main livelihoods of the villages (Fig. 2). Ethnic groups reside in the villages include Lao Loum (lowland Lao), Bru, and Phu Tai. The districts are among the poorest in the country, with average GPD per capita of 680 USD (6.53 million Kip) in Nong, and 763 USD (7.05 million Kip) in Phine in 2020. The districts also had among the highest malaria incidence in the country with a total of 403 cases in Savannakhet in 2020 (number of malaria cases reported by CMPE as of 2020). Study villages, including Phounmakmee (PMM) and Thathe (TT) in Nong, and Kaengsalang (KSL) in Phine, are located in remote and rural forested regions in the province whereby access to these villages is limited during the rainy season, particularly Kaengsalang. In these communities, forests were often described as mountainous areas with forest cover, forest-fringes, and sometimes forest farms.

### Respondents

In-depth interviews (IDIs) were conducted with forest goers who reported visiting the forest on more than 14 days/year and were aged 18 years or older. Purposive sampling was used to identify initial forest goers and snowball sampling was used to identify more forest goers in the villages. Consenting participants were asked about forest-related activities, experience with malaria, use of prevention measures, and their perceptions of prophylaxis. Additional in-depth interviews were conducted with local healthcare workers, community leaders, and policymakers in the villages or at their place of work. Purposive sampling was used to recruit stakeholders who were asked further about current and future approaches to address forest malaria and implementation of malaria interventions. The total number of respondents (forest goers and stakeholders) was a result of diversity sampling (forest-goers who undertake diverse activities and stakeholders occupying a variety of positions in villages and the health system) and reaching theoretical saturation, whereby no further new information was forthcoming from subsequent interviews.

### Data collection

In-depth interview guides were developed (Additional file 1, Additional file 2, Additional file 3, Additional file 4) for each type of respondent based on the initial themes from a recent qualitative study on forest going and malaria risk in Cambodia [17]. The guides were initially designed in English and translated into Lao by a national Lao speaker and field researcher (SI). They included key topic areas and a list of suggested questions, and were designed to be used in a flexible and iterative manner: interviewers would be reactive to the responses and probe or ask follow-up questions to elicit the information on specific topics emerging during the interviews. During development, the translations of the topic areas and suggested questions were discussed and checked with the field researcher who was also trained on how to conduct an interview using the guide. The guides were then piloted during field visits prior to data collection and with the first recruited study respondents to check for any miscommunication and revised as necessary. Meetings were conducted regularly between the field researcher and social scientists to debrief the interviews and supervise data collection.

The interviews were conducted between October and December 2020 in Laotian language; assistance was sought from local health workers present at the study site to assist in Bru translation when respondents felt that s/he could not express their thoughts fully. Interviews with forest goers were conducted in private in quiet locations at communal spaces within the villages, including a primary school or outside a health centre. Interviews with local and provincial stakeholders took place at their places of work. One interview with a national policymaker was conducted online by two Thai researchers (MJ and PK). Interviews took about 30–60 min on average. Observations and field notes were taken throughout study activities in the villages.

### Data processing and analysis

After respondents gave their consent, interviews were audio-recorded and transcribed by the field researcher. Two authors (SI and MJ) translated the transcripts and detailed interview notes into English and discussed the translated content extensively to ensure accuracy of the interview data. Using QSR NVivo 12, the transcripts were coded line-by-line using inductive and deductive approaches using the codebook that was constructed based on a previous qualitative study in Cambodia [17] and initial research topics (also drawing on the multiple components of acceptability and the various levels of context [18] that are relevant to implementation of health interventions). During the process of coding, themes that emerged from the data were incorporated into the codebook. Three authors (SI, MJ and CP) discussed content of the codes to explore the themes shown below particularly on the characteristics of forest goers and the opinions about malaria prophylaxis. The map in Fig. 1 was created using ArcGIS Pro software version 2.5.0 (Esri). Service layers used included World Hillshade (Source: Esri, Airbus DS, USGS, NGA, NASA, CGIAR, N Robinson, NCEAS, NLS, OS, NMA, Geodatasystem, Rijkswaterstaat, GSA, Geoland, FEMA, Intermap, and the GIS user community) and World Terrain Base (Source: Esri, HERE, Garmin, FAO, NOAA, USGS, © OpenStreetMap contributors, and the GIS user community). National, provincial and district-level administrative boundaries were retrieved from GADM database of Global Administrative Areas version 3.6 (GADM, 2018). Forest extent of the year 2017 was extracted from Global Forest Change 2000–2020 dataset (Source: Hansen, UMD, Google, USGS, and NASA).

### Ethical review

The study was approved by the Oxford Tropical Research Ethics Committee (OxTREC reference no. 534-19) and the National Ethics Committee for Health Research (NECHR reference no. 024). All respondents provided written informed consent to participate in the study and for the interviews to be audio-recorded. Consent was obtained from villagers when they were recruited in their villages or at their home, and verbally confirmed before the interviews. For stakeholders, written informed consent was obtained before the interviews. The study team engaged and consulted with local stakeholders, including health facility staff and officials at district and provincial levels, and staff of CMPE throughout the study. Health centre staff and village leaders subsequently facilitated the meetings with, and recruitment of, forest goers in the villages and assisted with explaining about the study and its purpose in local languages.

## Results

### Demographic characteristics of respondents

A total of 16 interviews with forest goers were included from Phounmakmee and Thathe villages in Nong, and from Khaengsalang village in Phine. Most forest goers were male who combined farming with forest going. Additional 15 interviews were conducted with healthcare workers including nurses at health centre and district malaria staff, local community leaders at village and district levels, and policymakers at provincial and national levels. Characteristics of the respondents are summarized in Table 1.

**Table 1:** Demographic characteristics of respondents

Forest goers		
Sex	Female	1
	Male	15
Age	18–20	4
	21–30	7
	31–40	4
	41–50	1
	51–60	0
Marital status	Single	1
	Married	12
	Not specified	3
Number of household members	1–4	4
	5–8	9
	9–13	3
Education years	1–6	4
	7–12	1
	none	11
First language	Laos	5
	Bru	6
	Bilingual (Laos and Bru)	5
Stakeholders		
Sex	Female	6
	Male	9
Age	21–30	4
	31–40	3
	41–50	2
	51–60	6
Occupation	Healthcare workers	5
	Community leaders	5
	Policymakers	5

### Forest visits

Most forest goers (or their family) owned and/or worked in a farm in or close to the forest. Respondents described how male and female community members visit the forest with their family, friends, or neighbours for multiple forest activities, including collecting food and wild products (such as mushrooms and bamboo shoots) and caring for cattle, for which most villagers visited the forest or forest fringe on a daily basis. Some respondents reported hunting and logging in the forest, for which they visited for periods ranging from a few days to a week, once or twice a month, depending on how far they travelled. During overnight trips, male respondents described moving between well-known places in the forest that they had visited previously. Travel time to the forest depended on the distance from the forest to each village and how forest goers travelled there. The closest forest to Phounmakmi village was about 2 km and 3–4 km for Kaengsalang village. Respondents in Thathe village reported having to travel 10–20 km to reach Baan Thathe mountain. Most respondents described how community members lived in poverty and were motivated to make their living from the forest.

Most respondents reported owning and working in rice fields and cassava farms. Farm visits could range from a few days to up to a month. Visits were longer in the rainy season, during which both male and female members of the family and their children stayed at their “Thieng Na” (farm huts or farmhouses), where families rest during the day and spend nights at. Respondents described being busiest with their farm work during June and July and making frequent visits to the forest during August to October. After harvest in October, villagers mostly stayed home in the cold or dry season from November to January.

Apart from collecting bamboo shoots for their own use and digging other tree roots for sale, villagers were also hired to work in rubber and hardwood plantations owned by Vietnamese companies that produce paper. Some respondents reported working in rubber farms all year round, for which they worked at night to early morning from 3 to 9am. Respondents described earning about a half of the yields from rubber products from land owners, whose products were later transported to Vietnam for sale. Local stakeholder respondents were aware that villagers lacked economic resources and made their living from the forest, describing that their main income came from selling forest yields and logs to mobile merchants and companies. Community leaders also described that villagers travelled deeper into the forest for logging because desirable trees at the fringe were mostly already logged.

“The rice harvest only feeds them [villagers] for 4 months and in the other 8 months they make a living in the forest.”

IDI with community leader, female, TT, Nong.

*“I often work in farms and rice fields ... sometimes I go to the forest with my brothers … about 2 kilometers from our house. (Do you spend nights there?) I sleep at the farm house, sometimes for 1 day, sometimes 5 days, mostly in August to clean the farm … I go to the forest more often in September”*

IDI with forest goer, male 33-year-old, TT, Nong.

*“… a lot of people go to earn in the forest. I usually go to find food, like bamboo shoots, and hunt in the forest (Where?) at Ta-Perm mountain, with 2-3 friends or alone sometimes. I go about 2-3 times a week … sometimes I go during the day, sometimes at night … for 2-4 nights during which I move around in the forest to find food and animals. (Where do you sleep?) I sleep in a cave or on palan-hin [stone terrace] … and use pa-bad [rubber cloth] to cover the net. I always use a mosquito net. Without it I cannot sleep.”*

IDI with forest goer, male 40-year-old, KSL, Phine.

*“I usually raise cows and farm in rice fields. I went to the forest to find wood to build [my] house … and find food near the river. (Is it far?) About 1 kilometer to the river, 2 to the forest with wood. (Did you spend nights there?) Last time I slept there for 3 nights. I spent 4 days finding wood and I took it back to the house that time … but usually I go to the forest during the day, only during logging did I stay at night. (How do you sleep?) I use the logs to make a shelter with covers. (Do you use mosquito nets?) No, I sleep on the ground. (Don’t you get mosquito bites?) Yes, but I can still sleep … the net was at the farm with my wife, I did not take it with me to the mountain.”*

IDI with forest goer, male 40-year-old, TT, Nong.

### Experiences of malaria, its prevention and treatment

Most forest goers were concerned about malaria and recognized its symptoms from their past experience; chills, cold, and fever were often described as the symptoms of the disease. One respondent called malaria a chill fever (or “Kai-Nao-San” in Laos) and described severe fever related to a negative spiritual symptom (or “Phee-Kao”). Some forest goers reported that they never had malaria before and did not want to have malaria. Respondents described mosquito bites as the cause of malaria and protecting themselves from bites when visiting the forest or water sources in the village where there were many mosquitoes.

Wearing long-sleeved clothes was reported to be the key means to prevent mosquito bites for forest goers. Respondents described sleeping under bed nets at their village home and less frequently at their farm hut. They reported receiving ITN from a health centre and/or malaria unit in the district. Some respondents were aware of mosquito coils and repellents but described not being able to afford to buy them from local shops; a few reported disliking the scent or smoke. One respondent reported using an ITN when staying at his farm hut, and he brought a hammock net and mosquito repellent with him to the forest. The respondent also described villagers frequently making fire or lighting tobacco for smoking (or “Kok-Soob-Ya” in Laos), which they grew themselves or bought from a local market, both of which produce a strong scent and keep mosquitoes away. Some described that those who hunted in the forest usually did not bring mosquito nets with them. Health worker respondents reported advising forest goers to carry and sleep under ITNs which they provided during the forest visits; they also suggested that villagers purchase an additional bed net to use at home. Hammock nets were described by programme staff to be available among the military who were positioned in the forest or forest camps; the hammock nets were perceived to “take too much space” among the military staff and were not sufficient to distribute to the general population.

*“I wear long-sleeved clothes. It’s good, long pants, hats and those things … (Do you use a mosquito repellent?) I never heard that they sell repellent here … (What about a coil?) Vietnamese gave me the coil … I did not buy it, we worked together in the rubber farm (When was this?) Just this year.”*

IDI with forest goer, male, 22-year-old from PMM, Nong.

*“(Do you use repellent?) No, I did not … I did not buy it so I did not use it. Mostly long-sleeved clothes, mosquito nets, … and light a fire. I also want [repellent] but cannot afford to buy it.”*

IDI with forest goer, female, 18-year-old from PMM, Nong.

Village health volunteers and health centre staff were described to be the main sources of information about malaria and the primary point of care for villagers in the area, especially those who lived far from the health centre or town. Some forest goers reported being familiar with getting a malaria test at the health centre and some received information about malaria from the nurse and district staff. They described also visiting the closest health centre for malaria treatment and for other treatment if they continued to have fever or other illnesses.

Health centre staff in the villages reported seeing up to fifty patients in a month with febrile illnesses, including colds, scrub typhus (“Khai Meng Daeng”), dengue (“Khai Yoong”), and malaria. They described that villagers would normally not visit the health centre when their symptoms were mild: they would “leave it to get better by itself” or treat themselves with traditional methods from local beliefs. One health centre staff gave an example of childbirth whereby the mothers disliked staying at the health centre after giving birth, although they were advised to rest for 24 h after delivery. Respondents described that most patients who visited them usually had severe symptoms, for example, from cerebral malaria described as “goes to the brain already”, and that it was difficult to treat them at the health centre. Patients with severe conditions needed to be referred to better equipped district or provincial hospitals; a process which the local staff perceived to be too slow.

Healthcare workers described getting their knowledge about malaria from their training and past experience. Most staff identified “PF” (*Plasmodium falciparum*) and “PV” (*Plasmodium vivax*) as different types of malaria. Some staff also mentioned asymptomatic malaria when patients without symptoms were sometimes tested positive for malaria using RDT by village health volunteers (VHV) or microscopy by malaria unit staff. Apart from giving information to their patients when they visit the health centre, respondents also described visiting villagers at their home in the villages to inform them about malaria and the importance of sleeping under ITNs once or twice in a year. Respondents also reported meeting fewer malaria patients in Nong district, from eighteen positive cases in 2019 to less than ten cases in 2020.

### Addressing forest malaria and approaches to malaria prevention and control strategy

#### Challenges to malaria service provision and implementation

When asked about challenges for malaria prevention and control in the area, policymakers highlighted that forest malaria is difficult to address because of the poverty the targeted population is living in, or without addressing poverty and their living conditions. High malaria transmission areas in southern Laos were identified: Attapue, Khammouane, Pakse, Salavan, and Savannakhet. Malaria programme and provincial staff described constraints targeting local residents in these areas whose livelihoods were close to the forest and at-risk mobile populations relocating to work in agricultural and mining companies. Staff were concerned that this group of workers were at increased risk because of their work in mines and plantations close to the forest. Staff were unable to provide malaria testing and treatment for these labourers or to access malaria case data from the private companies who often provided medical care for their own staff, giving an example that the high malaria prevalence in Nong district in 2018 was among Laotian and Vietnamese workers at a logging factory. Policymakers were also concerned about probable cross-border transmission among Laos military personnel stationed along the borders with Cambodia, China, and Vietnam.

*“There are a lot of challenges because malaria is very much related to poverty … and to eliminate malaria we need to understand the community first. The disease is in the forested area where people visit for wild products, hunting, logging, and digging tree roots. There is also a group who move from one town to other towns to work, and migrants who work for companies … Those areas are hard to access. It’s home to ethnic groups and people living at the foot of the mountains. They said malaria is from the forest but actually those who go to the forest live in the forest. So the mosquitoes are in their everyday lives.”*

IDI with policymaker, provincial level.

Lack of information on forest goers and on their use of ITNs was described as a challenge for programme staff to address forest malaria. They were also concerned about low awareness of the disease and use of prevention measures in the forest among at-risk populations, particularly ethnic groups in remote villages. Staff also observed that some households did not sleep under ITNs, some did not have enough bed nets for their family members, and some did not take the nets to use in the forest. Insufficient budget to provide LLINs for each household (1–2 LLINs per household) and low frequency of distribution (once a year), as well as inadequate options for prevention methods to be provided to this population, were also mentioned as limits to the programme’s capacity to control the disease.

*“Normally villagers stay at their farm … about 70-80 of them come to the health centre. When it’s drier and they have finished harvesting, about 100 patients come … up to 140 patients in a month. (Who visits you?) Mostly those who live close to the health centre. We don’t have patients from other villages. (Are there people who do not visit you?) There are some, for example, those who live far in Kaengsalang village do not come … because it’s difficult to travel, the road condition is poor. They need to come using a better route and we meet them at a meeting point.”*

IDI with healthcare worker, Phine.

Several challenges to provision of malaria services were highlighted by local health workers and programme staff. Respondents described that they could not reach the target number of malaria tests to actively find cases in the villages because some villagers have refused to do a blood test when they did not have symptoms. They were not able to conduct active case detection among workers in jewel mines in forested areas in the province, as their employers did not allow for the testing services from the programme. Staff were concerned about delays in malaria treatment, especially for those who lived in remote villages with limited access to transportation and communication with public health staff in the district. They were unable to effectively monitor treatment adherence, particularly among patients who lived in those villages or were highly mobile. They reported that primaquine could not be prescribed to patients when G6PD deficiency testing was not available at the local level due to fear of haemolysis. Furthermore, they were concerned about the risk of drug resistance in the areas as mobile workers, military staff, and those who illegally crossed borders might not always have access to public health services, and thus received counterfeit anti-malarials with which treatment was sub-optimal.

#### Approaches to improve malaria prevention and control strategy

Timely access to malaria services and increasing awareness of the disease among target populations were emphasized as important approaches to address forest malaria. Policymaker respondents reported that the patient referral process by VHV and health centre staff to hospital should be shortened. They described that more provision of information and health education about malaria are needed among at-risk groups. This includes how malaria is caused, how to prevent from catching it and what the negative consequences of infection are. Two programme staff also identified the need to provide more vector-control and prevention measures, including mosquito repellent, ITNs, hammock nets, and indoor residual spraying (IRS).

To improve adherence to anti-malarial treatment, one policymaker respondent described the need to pilot G6PD quantitative testing in local health facilities in high burden areas, such as Sekong and Attapeu. The respondent also identified the need to strengthen case surveillance and management by VHVs to improve treatment quality: cases needed to be recorded and reported, and monitor prohibited use and sale of anti-malarials at the local level. At the provincial level, one programme staff suggested that polymerase chain reaction (PCR) should be implemented in addition to current use of microscopy to improve the precision of malaria diagnosis. Training of, and support for, local health providers were considered essential in order to implement these interventions.

Areas of new studies and future research were identified by policymakers. Respondents described programme monitoring and evaluation, and empirical evidence-based implementation as significant to improve the programme’s current and future interventions. One respondent also suggested that more behavioural studies should be conducted to evaluate the use of ITNs among targeted populations for the programme to better understand the extent to which forest goers used ITNs when they visited the forest. One provincial staff also suggested to evaluate the long-term efficacy of the treated nets to explore whether the vector is insecticide-resistant. A few programme staff suggested use of technology, such as GPS, to track at-risk populations and identify malaria hotspots in the active foci areas. They described that the evaluation of these interventions and novel tools would help them devise a better strategy to achieve elimination by the target year.

### Prospects for prophylaxis

#### Perceptions of malaria prophylaxis

Malaria prophylaxis was generally not recognized among forest goers and local stakeholders in the study areas: respondents reported that they have never taken (or seen anyone taking) anti-malarial tablets for prevention. Forest goers were positive in their response when asked hypothetical questions about their willingness to take preventive medicine. One respondent described that he would like to protect himself from malaria if prophylaxis were available free-of-charge. A few respondents described preferring injection to taking tablets for prevention. Most respondents were also familiar with vaccination in children at the health centre. One respondent described that he was familiar with receiving treatment with injection from the health centre when he had a fever or stomach ache. The respondent also perceived that taking “too many tablets” would badly affect his health. In response to a hypothetical question on the preferred number of tablets, two respondents thought two tablets per month was a good amount of medicine to take for prevention.

“If there is a medicine to take to prevent, [we] would feel more confident when going to the forest … not scared of malaria”.

IDI with healthcare worker, PMM, Nong.

*“If there is a protection medicine, I would like to take it. (Where would you like to get it?) The health centre. (Do you prefer a tablet or a vaccination?) I am familiar with taking tablets … I don’t like the pain from injection.”*

IDI with forest goer, male 40-year-old, KSL, Phine.

*“I never heard of it [prophylaxis] before. Very good if we have that … but I also think there should be more impregnated nets because the villagers only received one once a year … sometimes once every two years.”*

IDI with community leader, TT, Nong.

*“(Would you be concerned about taking prophylaxis?) Not concerned, I can take it if there is one. Very good if there is a medicine for prevention but I have never heard about it before. (How often would want to take it?) I would take them during the peak season or before I might get infected … when there are still water sources and mosquitoes are growing … before the transmission gets high … how much I take should depend on what the program prescribes.”*

IDI with healthcare worker, KSL, Phine.

Concerns about potential side effects of prophylaxis were highlighted by local healthcare workers and community leaders; they were concerned whether the medicine would have side effects from long-term use. Problems with the kidneys and skin discolouration were mentioned as examples of negative effects medicine could have on health. Health workers in Nong district also described experiences of taking anti-malarials from a previous intervention (mass drug administration (MDA), three years prior) among villagers, during which there were complaints about its side effects, such as sore body, and feeling frustration from taking the pills. Health centre staff and village chief were also unsure whether the medicine helped villagers with malaria or not.

Although prophylaxis for malaria was not available in Savannakhet, it used to be recommended for public health staff to take anti-malarials before visiting endemic areas in the past. Provincial staff reported that the prevention option did not continue as malaria declined in the area. Chloroquine and mefloquine were respectively described as being used for prophylaxis by local government staff and foreign officials. These regimens were reported as being first-line treatments for malaria before 2005. They also described that the option was not implemented among the local population because the measure was not evaluated for its effectiveness; the programme was not aware whether the medicines work to protect the staff or not. Staff commented that their current approaches for malaria prevention put more emphasis on vector-control measures, such as ITNs; provision of prophylaxis for local populations had been discussed (among the programme’s therapy working group), but not perceived at the time to be a viable alternative intervention.

#### Essential considerations for prophylaxis as a strategy

Several factors were described by policymaker respondents to be essential to make a decision about prophylaxis as a strategy: choice and efficacy of prophylaxis, adherence to and frequency of the regimen, and estimated cost and availability of the medicine. For prophylaxis to be considered as a strategy, policymakers suggested that there should a pilot study to explore the efficacy to better understand the extent to which it can prevent malaria among forest goers. A controlled trial comparing treatment and control groups was suggested “to prove whether prophylaxis can prevent malaria a hundred percent”. Respondents also suggested that the choice of regimen should consider a higher quality anti-malarial than the previous regimen i.e. chloroquine that was used by government officials.

Adherence to prophylaxis was also a significant consideration. Programme staff pointed out that some villagers or forest goers may not take the medicine when they are not sick. Policymakers expressed similar concern to forest goers related to an appropriate frequency of the doses: if the dosing is frequent or complex, the respondents suggested that villagers would likely forget to take them. Taking prophylaxis once a week or once a month was suggested to be possible among the local population whose concerns depend largely on the number of tablets and their side effects. Staff also suggested that the intervention needs to give the villagers the confidence that the medicine can protect them from malaria for them to adhere to it. However, some were concerned that taking preventive medicine might discourage other preventive behaviours if forest goers felt that the medicine already protected them from malaria. In addition, policymakers raised concerns about possible inappropriate drug intake among forest goers, and whether it can potentially impact drug resistance when an anti-malarial is used prophylactically for a long period of time.

“If they go to the forest every day, does it mean they have to take the medicine every day? They may forget or they may not want to take it on a daily basis.”

IDI with policymaker, provincial.

Financial cost of prophylaxis was also a crucial component of the intervention’s feasibility. If prophylaxis were proven to be efficacious, programme staff raised questions about how the medicine should be funded. They were also concerned about its accessibility; if the medicine were not subsidized, how much the anti-malarials should cost and whether forest goers could afford them. Programme staff suggested that the programme needed to consider its capacity to pay for the medicine and whether the medicine is worth the cost, giving the example of the high price of Pyramax—a newly introduced anti-malarial treatment in the country.

Nevertheless, policymakers suggested that prophylaxis could be considered as a strategy to implement in high burden areas in the country. One respondent also suggested comparing the efficacy of prophylaxis to that of MDA, an intervention which was perceived to be effective only during the implementation period. Lessons learnt from other countries, WHO guidelines, and scientific research, on use of prophylaxis were described as strong evidence for policy-making considerations. Policymakers also noted that the intervention needs to be assessed for its feasibility to create context-specific lessons for possible scale-up.

## Discussion

The findings outline the nature of forest going in endemic villages of Savannakhet Province, Lao PDR and perceptions of malaria prevention and health services among forest goers: respondents were at an increased risk from their forest visits and had minimal prevention measures against mosquito bites in the forest or forest farms. Interviews with stakeholders highlighted their views on several challenges for targeting this at-risk population and approaches to address forest malaria in southern Laos. Their opinions about malaria prophylaxis as an intervention revealed several essential considerations for its feasibility.

### Profiles of forest goers and their malaria risk

The findings indicate that malaria endemic villages in Savannakhet are home to a mixed group of forest goers: farmers who visit the forest as their livelihood or employed to work in forest farms, and mobile workers, such as labourers and miners, cross-border migrants, and military officials. Their malaria risk may differ according to their activities and time of exposure. For villagers in these areas, several studies have identified multiple malaria risk factors, including staying overnight in farm huts, night-time working and sleeping in the forest, spending time outdoors at home, staying in houses close to mosquito breeding pools, and insufficient use of ITNs in endemic villages in Sekong [19], Xepon [20], and Attapeu [21]. In Vietnam, high malaria risk was reported during periods when activities took place outside of bed nets, such as evening drinking gatherings, fishing, logging in the forest, outdoor television watching [22], and living in wooden or bamboo houses [23]. For mobile workers, who were mostly Laotian and Vietnamese adult males of private companies, and Laotian military staff, their mobility has posed a challenge for the programme to reach them for malaria control measures [24]. Different strategies should be implemented to target these population groups for malaria interventions. Stronger communication with VHVs and health centres are crucial to reach local forest goers, while coordination with private companies or other governmental organizations may be necessary to reach these mobile workers at risk.

Forest goers in these villages used minimal measures for malaria prevention in their daily lives: mostly long-sleeved clothes, lighting fires, and occasionally ITNs were reported. Their use of malaria prevention was more rudimentary compared to other neighbouring countries, including Cambodia [17], Thailand [25], and Vietnam [26], where the use of long-lasting insecticidal hammock nets (LLIHNs) and mosquito repellents were described. A survey among military personnel in Champasak and Attapeu provinces also reported low awareness of malaria risk and use of preventive measures, and emphasized that sufficient supply of mosquito repellent and coils should be provided [27]. This suggested that interventions targeting forest goers and those who were at increased risk in the forest needs to consider providing additional prevention measures to better equip them with protection in forest settings.

### Access to malaria services and quality healthcare in the community

Limited access to malaria and other health services was the main barrier to effective malaria prevention and control in the area, with delayed referral to treatment being common. In addition, villagers often opted for self-treatment and only visited health centres for severe conditions. Studies have reported varied factors that discouraged treatment-seeking behaviours in remote communities, including distance from home to health centre, road conditions, lack of transportation, low economic status and costs from healthcare visits [28], lack of social support and belonging to an ethnic group (or hill tribe) [29], unavailability of healthcare providers [30], and preference for traditional healers to treat fever symptoms [31]. Improving access to malaria services, as well as healthcare delivery in general, thus entails developing better infrastructure for transportation and communication in these communities.

Other febrile illnesses were important health concerns among forest goers and health providers in the communities. Dengue, leptospirosis and scrub typhus were reported as the most common treatable diseases in rural Laos and these could be treated by local health workers [32]. To sustain malaria services for detecting and treating the remaining cases, a recommendation was made for VHVs to provide an integrated package of services to meet the needs of their communities as malaria declines [33]; optimizing such community-based services could better address local health concerns and be more responsive to local epidemiology of those diseases.

### Reflections on malaria prevention and control

The provision of ITNs remains a central approach to vector-control measures and malaria prevention in Lao PDR. Nonetheless, programme staff described needing more evidence and evaluation studies to assess the use and effectiveness of ITNs and further develop other prevention measures. Previous studies have assessed the efficacy of ITNs in southern provinces of Laos and suggested the programme should evenly distribute ITNs and promote their use in remote villages to ensure good coverage [34, 35] and reduce malaria infection risk [36]. Indoor residual spraying (IRS) and use of repellents were also recommended as necessary vector-control measures among communities in forested borders between Laos and Vietnam [37]. These interventions should be considered alongside future provision of ITNs (and health education) in these communities.

Difficulties in reaching MMPs and plantation workers posed challenges to effective malaria case surveillance and management: programme staff were concerned about the efficacy of the anti-malarial to treat malaria and possibility of spreading drug resistance. This was compounded by the risk of MMPs and workers with unclear legal status receiving counterfeit or substandard anti-malarials from mobile drug vendors: fake artesunate was recognized as a major threat in Lao PDR and other GMS countries [38]. To tackle this problem, distribution of integrated packages for malaria prevention at multiple points of entry or mobility was recommended to the malaria programme [24]. Coordination with non-governmental organizations and civil society groups was suggested to ensure access to these measures among this population group.

Administration of G6PD testing and malaria diagnosis using real-time PCR were recommended to better understand the burden of vivax malaria and develop malaria elimination approaches. The need for G6PD testing was previously echoed by malaria programmes to administer a shorter primaquine regimen and address low adherence to anti-malarials in Laos PDR [39, 40], Cambodia and Thailand [41]. Furthermore, unavailability of G6PD rapid diagnostic kits at local health facilities may limit its implementation as patients would have to visit district or provincial hospitals to receive the regimen [42]. For these strategies to be realized, training of local health staff is required to roll out G6PD testing in endemic areas. Household-based strategies, including reactive case detection targeting the household members of index cases, was also suggested as an approach to address asymptomatic cases in low transmission settings [43].

### Prospects for prophylaxis and malaria elimination

In response to hypothetical questions, forest goers perceived the benefits of taking preventive medicine to protect themselves from malaria but were concerned about its long-term effects on their health. Concerns about side effects from anti-malarials were similarly raised in MDA participants in this region who were worried that the side effects would negatively affect their health and their ability to continue their farm or forest work [44]. For some villagers, taking anti-malarial tablets was perceived to be less beneficial compared to an injection. This reflects a broader observation across South East and East Asia whereby intravenous fluids (IV) are commonly used for pain relief with saline (as a placebo) or sometimes antibiotics [44], and for anti-malarials in Cambodia [45]. If the regimen comprises a complex dosing or multiple tablets, prophylaxis may be less accepted among forest goers and they may perceive the medicine to be harmful to their health.

In Nong district, some forest goers and local stakeholders were aware of asymptomatic malaria, which may have been partly influenced by previous MDAs in the area [46]. From its implementation, the community’s recognition of malaria as a health concern and awareness of asymptomatic malaria have resulted in higher participation coverage in the villages [47]. Provision of other basic care and being responsive to their needs and preferences were also crucial for such community-based interventions to be taken up and accepted among community members, particularly in remote communities with limited access to healthcare (and other economic) resources [48]. A recent review of end-user perspectives on preventive anti-malarials also suggested that uptake of, and adherence to, implementation of such preventive therapy required trust building and multiple stakeholders engaging throughout its implementation [49]. Providing information on asymptomatic malaria is beneficial to raise awareness of the disease among community members [50]. This information may also provide a comprehensive understanding of malaria infection and different ways to protect themselves from the disease, including prophylaxis and how it functions as a prevention option.

Concerns about cost of the prophylactic regimen to the malaria programme were described as the major challenge for policymakers in this region [51]. Although malaria case detection and surveillance have been significantly improved in Lao PDR, the programme faced difficulties in maintaining the supervision, equipment and financing for those activities and for VHVs [33]. In addition, the need for evidence of the efficacy of prophylaxis highlighted their concern about adherence (and drug misuse) among the target population, implementation challenges, and perceived consequences for multi-drug resistance. Similar concerns were raised about MDA as an elimination strategy, for which lack of data on its effectiveness and lack of support from the WHO for the intervention were influential to the programme’s decision-making [52]. Strengthening the evidence from piloting interventions in the Laos context was thus essential to their consideration of its feasibility.

A set of considerations for implementation of anti-malarial chemoprophylaxis among forest goers were identified in Lao PDR (Table 2). While it was found that forest goers were hypothetically willing to use prophylaxis for malaria prevention; there were pending considerations for its feasibility in high burden areas. The findings also emphasize current challenges of targeting mobile and migrant populations and providing timely access to malaria preventive and treatment services. For Savannakhet, implementation of multiple interventions as an integrated strategy was recommended to ensure universal access to malaria diagnosis, treatment, and prevention [53]. Building on the programme’s achievements in fine-tuning its surveillance system and addressing recent outbreaks, steps have been taken to implement a package of strategies to complement its core interventions targeting forest goers [54]. As Lao PDR and other GMS countries work towards malaria elimination, strengthening the capacity of local health workers in case management and provision of universal health services are critical to reach this population group and optimize future interventions.

**Table 2:** Main considerations for implementation of malaria prophylaxis among forest goers as a strategy

Main policy implications	
Prophylaxis as a part of malaria elimination strategy	1. Employ different strategy that includes prophylaxis and other malaria prevention measures to target at-risk populations; working with VHVs to reach community members and coordinating with private sector/other government to reach workers
	2. Recommended to pilot prophylaxis in high burden area
Choice of regimen	3. Evidence of efficacy and funding sources for anti-malarials are key determinants 4. Less complex and minimum number of tablets are preferable to avoid perceived side effects
Delivery of prophylaxis and provider	5. Significantly constrained by lack of transportation and communication barriers in remote communities 6. Strengthening capacity of local health workers and providers are crucial to monitor adherence and follow-up of patients
Messages about prophylaxis	7. Knowledge about asymptomatic malaria could be beneficial to create a better understanding of prophylaxis and encourage its uptake as a prevention therapy

### Strengths and limitations

This is the first study to use qualitative research methods to explore prospects of prophylaxis for malaria control and elimination in Lao PDR, and it adds to previous studies on malaria risk in southern Lao PDR [28, 46, 55]. The data collection was limited by using one trained researcher to conduct the interviews due to travel restrictions for other researchers during the COVID-19 pandemic. The data elicited from IDI respondents are reported data and might be subject to desirability bias. However, observations and interviews with multiple groups of informants provided additional information on the context of forest going and sensitive topics, such as logging and illegal activities. The interviews were conducted in Lao language including those with other mother tongues (Bru); assistance was sought from local staff who spoke the language and provided translation of specific terms for the respondents. Almost all forest goer respondents were male, which reflects how male community members are most engaged in forest activities. They were diversely drawn from endemic villages close to the Lao PDR-Vietnam border. Stakeholder interviews were conducted with multi-level respondents who were engaged in malaria control at village, district, provincial, and national levels.

## Conclusion

In ethnically and geographically diverse villages of Savannakhet Province, Lao PDR, awareness of malaria risk prompts forest goers to protect themselves, albeit sub-optimally using available preventive measures. To reach this population group, malaria interventions should be tailored to ensure access to necessary prevention measure and quality malaria services among forest goers, including mobile and migrant populations. Among policymakers, choice and cost of anti-malarial, particularly its efficacy and source of funding, are key considerations for the feasibility of malaria prophylaxis as an intervention. Implementation of prophylaxis is affected by a lack of transportation and communication barriers in remote communities. Acceptability of prophylaxis among forest goers is also influenced by the complexity of the regimen including number of tablets and timing of doses. Adding prophylaxis to existing malaria control activities requires strengthening the capacity of local health workers. Ideally, this would be part of an integrated approach that includes strategies to address the other febrile illnesses that forest goers describe as priority health concerns.

## Supplementary Information

**Additional file 1**. Forest goer IDI guide.

**Additional file 2**. Healthcare worker IDI guide.

**Additional file 3**. Community leader IDI guide.

**Additional file 4**. Policymaker IDI guide.

## Data Availability

The data on which this article is based cannot be shared publicly due to confidentiality of the individuals who participated in the study. The data are available upon reasonable request to the Mahidol Oxford Tropical Medicine Research Unit Data Access Committee (datasharing@tropmedres.ac) complying with the data access policy (https://www.tropmedres.ac/units/moru-bangkok/bioethics-engagement/data-sharing/moru-tropical-network-policy-on-sharing-data-and-other-outputs) for researchers who meet the criteria for access to confidential data.

## Abbreviations

CMPE: Center of Malariology, Parasitology, and Entomology
GMS: Greater Mekong Subregion
G6PD: Glucose-6-phosphate dehydrogenase
IDI: Individual in-depth interview
IPT: Intermittent preventive treatment
IRS: Indoor residual spraying
ITN: Insecticide-treated mosquito nets
LLIN: Long-lasting insecticidal net
LLIHN: Long-lasting insecticidal hammock net
MDA: Mass drug administration
PCR: Polymerase chain reaction
PHD: Provincial health department
RDT: Rapid diagnostic test
SMC: Seasonal malaria chemoprevention
VHV: Village health volunteer
