# Supplementary material for: Forest malaria and prospects for anti-malarial chemoprophylaxis among forest goers: findings from a qualitative study in Lao PDR
Source: Malar J. 2022 Jan 5;21:8. doi: 10.1186/s12936-021-04027-z (PMC8727080; doi:10.1186/s12936-021-04027-z)
Supplement: Supplementary file 1 — Additional file 1. Forest goer IDI guide. [file 12936_2021_4027_MOESM1_ESM.docx]

**Interview guide for forest-goers (Laos/Thailand)**

| **Instructions:**   - Follow the informed consent procedures - If consent is given, audio record the interview - This interview guide is to be used in a flexible manner - The aim is to collect in-depth information from the respondent - The left-hand column lists the topic of interest - The right-hand column contains a list of suggested questions and probes. - It is not necessary to ask all these questions in the order listed; these provide ideas to prompt the respondent to talk about the topic of interest - Use a flexible approach and probe as necessary: add extra questions depending on the responses - You do not need to follow the order of topics below; follow the responses/flow of the conversation. |
| --- |

| **Topics** | **Possible questions and probes** |
| --- | --- |
| **Opening** | Hello, my named is…   - Read out the information sheet - Obtained informed consent |
| **Socio-demographic information** | - Age group - Gender - Village - Employment/livelihood - Languages spoken - Literacy/education (number of school years) |
| **Forest visits** | - For what reasons do you go to the forest? - What do you do there? - How far away is the forest from where you live? - Who do you go to forest with? Do you ever take any family members? - How long do you visit for each time you go? - When do they go there? How often? - Do you move around in the forest or do you stay in one place? - Do you go to different places for different reasons? Please explain… - Do you go to different places at different times of year? - When are you at home most? What seasons? |
| **Sleeping arrangements in forests** | - Where do you sleep when you are in the forest? - Can you describe it (are there walls? overhead coverage? on the floor?) - Do others sleep in the same place? - Do you always sleep in the same place? If not. Where do you move to? Is it the same type of building? - Does everyone use a hammock or sleep under a net? - Where do you get the hammocks or nets from? How often do you replace them? Do you treat them? - What about if you go to a new place? Do you encounter other groups? |
| **Personal malaria-related experience** | - Have you had malaria before? - How did you know it was malaria? Was your blood tested? By whom? Using what? - Tell me about the last bout of malaria that you had… - When did you get ill? - Who diagnosed the disease? - Where do you think you got malaria from in the past? |
| **Experiences of malaria diagnosis** | - Have you ever had a blood test for malaria? - What type of test was it? - Have you ever been tested and got a negative result when you were sick? - What did you think about this? |
| **Experience of antimalarials** | - What are your experiences of taking malaria drugs in the past? - Can you tell me where you obtained/got the drugs? What were the drugs called? - Did you have any side effects? Can you describe them? - Would you take them again in the future? - Would you be willing to take these tablets if you weren’t sick but it could prevent malaria? - Do you use treatments from other sources for malaria? |
| **Malaria-related knowledge** | - What do you know about malaria? - How is the disease caused? - Do you think anything you do puts you at risk of getting malaria? - Do you know of any places in the area where there is a high risk of malaria? - Do you think it’s possible to be infected with malaria but not have any symptoms? - How did you distinguish malaria from other diseases? - How did you get your information about malaria? - Have you heard about different types of malaria? What can you tell me about that? |
| **Personal protection** | - What do use to prevent from getting malaria when you are in the forest? (unprompted) - Prompted:  1. Insect repellent? 2. Coils? 3. Bed net? 4. Hammock net? 5. Fire? 6. Antimalarials? 7. Other (specify)? |
| **Understanding of other disease prevention** | - What are the ways that you prevent from getting diseases yourself? - To prevent malaria, do you use bednets/hammock nets? Coils? Long sleeves? Or anything else? If not why? - Do you know where to get nets/hammocks etc.. from? - What do you know about vaccination? - Have you ever been vaccinated before? When? What for? - Do you think that you will never get that (or other diseases) after being vaccinated? Do you still need to protect yourself from that disease? Has anyone ever explained vaccination to you? If so, who and can you remember what s/he explained? Have you seen posters about vaccination? Do you remember the message of the poster? - Did your children get vaccinated? - Do you prefer injections or medicines to prevent (or cure) diseases? Can you compare these 2 ways? - If you were to take medicine to prevent from getting malaria, where would you prefer to get it from? Why? - For how long would you be willing to take medicine to prevent from getting malaria if it was once a week? Once a month? |
| **Closing** | - Do you have anything to add or any questions? |
